# Heterogeneous tempo and mode of evolutionary diversification of compounds in lizard chemical signals

**DOI:** 10.1002/ece3.2647

**Published:** 2017-01-29

**Authors:** Roberto García‐Roa, Manuel Jara, Pilar López, José Martín, Daniel Pincheira‐Donoso

**Affiliations:** ^1^ Departamento de Ecología EvolutivaMuseo Nacional de Ciencias Naturales – Consejo Superior de Investigaciones Científicas (MNCN‐CSIC)MadridSpain; ^2^ Laboratory of Evolutionary Ecology of AdaptationsSchool of Life SciencesJoseph Banks LaboratoriesUniversity of LincolnLincolnUK

**Keywords:** animal communication, chemosensory, disparity, lizards, pheromones, sexual selection

## Abstract

Important part of the multivariate selection shaping social and interspecific interactions among and within animal species emerges from communication. Therefore, understanding the diversification of signals for animal communication is a central endeavor in evolutionary biology. Over the last decade, the rapid development of phylogenetic approaches has promoted a stream of studies investigating evolution of communication signals. However, comparative research has primarily focused on visual and acoustic signals, while the evolution of chemical signals remains largely unstudied. An increasing interest in understanding the evolution of chemical communication has been inspired by the realization that chemical signals underlie some of the major interaction channels in a wide range of organisms. In lizards, in particular, chemosignals play paramount roles in female choice and male–male competition, and during community assembly and speciation. Here, using phylogenetic macro‐evolutionary modeling, we show for the very first time that multiple compounds of scents for communication in lizards have diversified following highly different evolutionary speeds and trajectories. Our results suggest that cholesterol, α‐tocopherol, and cholesta‐5,7‐dien‐3‐ol have been subject to stabilizing selection (Ornstein–Uhlenbeck model), whereas the remaining compounds are better described by Brownian motion modes of evolution. Additionally, the diversification of the individual compounds has accumulated substantial relative disparity over time. Thus, our study reveals that the chemical components of lizard chemosignals have proliferated across different species following compound‐specific directions.

## 1 Introduction

Animal communication influences the trajectories of social, ecological, and phenotypic evolution across multiple levels of biodiversity, from the sexes to the complexity of assemblages (Smith, 2013). Not surprisingly then, the quantitative study of the drivers, rates, and directions of diversification of signals employed by animals to engage in social and sexual communication has been the focus of an increasing stream of studies, which have flourished with the development of phylogenetic approaches designed for comparative analyses (Chen, Stuart‐Fox, Hugall, & Symonds, 2012; Derryberry et al., 2012; Mason, Shultz, & Burns, 2014; Ratcliffe & Nydam, 2008). As a result, the implementation of multiple programs of research investigating the adaptive evolution of signals across broad ranges of species varying extensively in their “strategies” for production and delivery of signals, and in the environmental pressures (i.e., sources of selection) shaping them, has contributed to accelerated advances in our understanding of the evolutionary dynamics of animal communication at larger spatial and taxonomic scales.

Given that animal species employ a broad diversity of phenotypic traits during communication, systems of production and delivery of signals are known to be shaped by multiple extrinsic (e.g., resource availability, population density, sex ratios) and intrinsic (e.g., phylogenetic inertia) factors. Indeed, both sexual and natural selection can often operate in coordination or antagonistically to shape the same signal. For example, while signal expression can positively correlate with the “genetic quality” of the signaler, the expression of the signal itself can compromise the expression of other energetically costly traits with strong effects on fitness (Irschick, Briffa, & Podos, 2014; Losos, 2009; Simmons & Emlen, 2006).

As a result of the accelerated development of phylogenetic methods for comparative analyses of trait evolution, a stream of studies has investigated the diversification history of signals in animals. However, the overwhelming majority of such studies have been focused on visual and acoustic signals (Gingras, Mohandesan, Boko, & Fitch, 2013; Huang & Rabosky, 2014; Santana, Alfaro, Noonan, & Alfaro, 2013; Wilkins, Seddon, & Safran, 2013). In contrast, comparative studies of chemical signals remain fundamentally ignored in most groups of organism (Kather & Martin, 2015; Symonds & Elgar, 2008).This gap of knowledge could hinder the emergence of new ecological and evolutionary hypotheses in the context of multimodal communication (Faria et al., 2014; Stacks & Salwen, 2014). Therefore, investigating the evolutionary tempo and mode of chemosignal diversification along the phylogenetic history of lineages that rely on these forms of communication is a major pending step to strengthen our overall understanding of the evolutionary dynamics of communication.

Research on chemical communication has highlighted the key role that chemosensory systems play in species interactions, niche adaptation, speciation, and extinction (Amo, Galván, Tomás, & Sanz, 2008; Apps, Weldon, & Kramer, 2015; Bacquet et al., 2015; Martín & López, 2015; Steiger, Schmitt, & Schaefer, 2011). However, techniques aimed to investigate communication at the chemical level are analytically demanding, and thus, ongoing advances in this field have been slower than research on other signals, such as visual and acoustic (Touhara, 2013). Despite these difficulties, some accelerated improvements in the development of technologies and methodologies for chemical analyses have inspired an increasing interest in exploring an expanding range of questions around the ecology and evolution of chemical interactions (Baeckens, Driessens, & Van Damme, 2016; Ding et al., 2014; Johnston & del Barco‐Trillo, 2009; Martín & López, 2015; Symonds & Elgar, 2008; Wyatt, 2014). These advances have made it increasingly more feasible to explore in detail the evolution of chemical signals and their multiple compounds across different species, and across multiple individuals within species. However, studies investigating the macro‐evolutionary diversification of the chemical components of communication remain fundamentally neglected (Steiger et al., 2011; Symonds & Elgar, 2008; Weber, Mitko, Eltz, & Ramírez, 2016).

In reptiles, in particular, chemosensory systems have been shown to play paramount roles in social and sexual interactions (Labra & Niemeyer, 1999; Martín & López, 2015; Mason & Parker, 2010; Pincheira‐Donoso, Hodgson, & Tregenza, 2008). In fact, phenomena as important as female mate choice mechanisms are thought to rely more heavily on chemical than on other forms of signaling among lizards (Kopena, Martín, López, & Herczeg, 2011; López & Martín, 2012; Martín, Moreira, & López, 2007). In these reptiles, a number of studies have failed to identify evidence revealing a role for quantitative traits biasing mating success during female mate choice (which has consolidated the view that sexual selection in these animals takes place via male–male contests; Olsson, Madsen, & Møller, 1998). In contrast, accumulating evidence suggests that this mechanism is fundamentally mediated by chemical signals (i.e., chemical compounds or/and a mixture of them; Martín & López, 2014, 2015) from secretions produced by follicular femoral and precloacal glands (Cooper, 1994; Escobar, Escobar, Labra, & Niemeyer, 2003; Flachsbarth, Fritzsche, Weldon, & Schulz, 2009; García‐Roa, Cabido, López, & Martín, 2016; García‐Roa, Carreira, López, & Martín, 2016; Martín & López, 2014). Indeed, recent literature confirms that both natural and sexual selection are affected by these secretions (López & Martín, 2005; Martín & López, 2006c; Martín, Ortega, & López, 2015; Martín et al., 2007). For example, studies focused on global warming have shown that the effect of different climatic variables alters the efficacy of chemoreception in lizards and, consequently, the fundamental basis of communication underlying population stability (Martín & López, 2013; Martín et al., 2015). Also, experiments conducted in males of European green lizards (*Lacerta viridis*) showed that females preferred to use areas scent‐marked by males with high proportions of vitamin E (Kopena et al., 2011). Similar female preferences for males producing “quality” secretions have also been reported in other species (Martín & López, 2015). Therefore, the study of chemosignal evolution has emerged as a vital perspective to push forward our understanding of species and trait diversification.

In this study, we present the first empirical study investigating the macro‐evolutionary diversification of chemical compounds found in femoral and precloacal secretions produced by lizards to engage in social communication. Among reptiles in general, species of the superfamily Lacertoidea have offered classical model systems shaping our understanding of chemical communication, and thus, the chemical profiles of their secretions have been routinely described in the refereed literature (Martín & López, 2014). In fact, lacertoid lizards have been the subject of the greatest number of behavioral and chemical ecology experiments to date (Martín & López, 2014, 2015; Weldon, Flachsbarth, & Schulz, 2008). Consequently, this lineage provides an ideal point of reference to quantitatively characterize evolutionary variation of chemical traits underlying communication. Specifically, we investigate the evolutionary trajectories and rates of diversification of particular chemical compounds over time, by employing phylogenetic modeling of the relative proportion of each compound measured in the secretions of each species.

## 2 Material and Methods

### 2.1 Study species

We gathered a comprehensive dataset encompassing 20 lacertoid species for which the detailed chemical composition of their male chemical secretions has been profiled (reviewed in Martín & López, 2014). In this study, we added information for the chemical composition of the secretions of other five species for which these data remained unavailable (Table S1). The total sample of species we have employed for this study encompasses a broad diversity of environments, which captures a range of areas where selection is expected to operate in contrasting ways as a result of variations in climate and in the intensity of interspecific competition arising from coexistence with other lizard species (Cox & Temple, 2009). For the preparation of our species‐level dataset, we averaged values of relative amounts for chemical compounds taken from multiple populations per species if they were available (see references of Table S1).

### 2.2 Chemical compounds

We performed an exhaustive collection of data on the relative abundance of particular compounds found in femoral and precloacal secretions from the refereed literature as well as from samples directly collected and processed by ourselves. Lizard chemical secretions are highly complex and consist of multiple compounds. We focused on the following subset of chemicals given their identified role in ecological interactions and communication in these reptiles(Martín & López, 2014; Weldon et al., 2008): (1) cholesterol, a steroid, usually the most abundant compound found in lizard secretions, which is thought to play a role in “holding” and protecting other compounds (Escobar et al., 2003; Weldon et al., 2008). High levels of cholesterol have also been associated with dominance (Martín & López, 2007) and intersexual interactions (Martín & López, 2006b); (2) campesterol, a relatively common steroid in lizard secretions, particularly dominant or highly common in some lineages (e.g., *Psammodromus* and *Gallotia*, respectively). High levels of campesterol have been associated with signal quality (López & Martín, 2009; Martín & López, 2006a); (3) stigmasterol, a relatively common, but not abundant steroid that is believed to be acquired via ingestion of plants. This compound is associated with structural properties in secretions, as well as with healthy conditions (Othman & Moghadasian, 2011); (4) ergosterol (i.e., provitamin D_2_), a common steroid that acts as a metabolic precursor of vitamin D_2_, and believed to offer a reliable indicator of male healthy condition. Therefore, this compound has been seen to play a key role in mate choice (Martín & López, 2006c, 2008), making in particularly interesting given the difficulties to demonstrate mate choice in lizards based on quantitative traits (Olsson et al., 1998); (5) 9,12‐octadecadienoic acid (i.e., linoleic acid) is a unsaturated fatty acid, costly to obtain. It has been attributed important functions in metabolism, and thus, it might act as an indicator of male “quality” (Martín, Chamut, Manes, & López, 2011; Weldon et al., 2008); (6) α‐tocopherol (i.e., vitamin E), usually found in lizard species in high proportions. It is believed to have antioxidant properties, protecting other compounds in secretions (Brigelius‐Flohe & Traber, 1999; Wolf, Wolf, & Ruocco, 1998). Also, high levels of α‐tocopherol are linked to the quality of lizards, and therefore, it has been assigned an important role during competition over sexual mates (Kopena et al., 2011); (7) cholestanol, commonly found in lacertids, and thought to be related with healthy body condition (Weldon et al., 2008); and (8) cholesta‐5,7‐dien‐3‐ol, a steroid present in some lizard species, it is the precursor of vitamin D_3_. It has also been related to male quality, acting as a potential indicator of health condition (López & Martín, 2005; Martín & López, 2006b).

### 2.3 Chemical analyses of secretions

We analyzed chemical secretions produced by femoral glands of males of the species shown in Table S1. We employed traditional techniques based on gas chromatography (GC) methodology, by using a Finnigan‐ThermoQuest Trace2000 GC fitted with a poly (5% diphenyl/95% dimethylsiloxane) column (Supelco, Equity‐5, 30 m length × 0.25 mm ID, 0.25 μm film thickness) and a Finnigan‐ThermoQuest Trace mass spectrometer as the detector. We conducted splitless sample injections (2 μl of each sample dissolved in *n*‐hexane) with helium as the carrier gas, and injector and detector temperatures at 250 and 280°C, respectively. The GC process was programmed with an initial temperature at 50°C (10 min), and posterior increase in temperature until 280°C (at a rate of 5°C/min), and kept finally at this temperature for 30 min. Mass spectral fragments below *m*/*z* = 46 were not recorded. Initially, we identified secretion compounds by comparing their mass spectra with those in the NIST/EPA/NIH (NIST 02) computerized mass spectral library. Then, the confirmation of identifications was done by comparing spectra and retention times with those of authentic standards (from Sigma‐Aldrich Chemical Co.) when these were available. We did not consider impurities identified in the control vial samples.

The relative amount of each compound was determined as the percentage of the total ion current. Finally, we collated the compounds‐of‐interest amounts to generate the final data base.

### 2.4 Phylogenetic macro‐evolutionary analyses

To quantify the evolutionary diversification of the selected compounds, we employed phylogenetic macro‐evolutionary analyses based on a model‐selection approach. These analyses were performed on a time‐calibrated molecular phylogenetic tree for our focal lizards, extracted from Pyron, Burbrink, and Wiens's (2013) supertree for squamate reptiles (lizards and snakes).

We compared the tempo and mode of evolutionary diversification of the individual chemical compounds along the phylogenetic tree against a range of models that describe the directionality and speed of trait evolution during a lineage's history. We first compared four evolutionary models: a traditional Brownian motion model (BM), which describes a random walk of trait evolution along the branches in the phylogeny. This model describes increases in trait variance centered on the initial value at the root of the tree, and increasing with the distance from the tree root. An Ornstein–Uhlenbeck model (OU), which assumes that once traits have adaptively evolved, stabilizing selection pulls the trait values around an adaptive optimum for the trait. An early‐burst or “niche‐filling” model, which describes exponentially increasing or decreasing rates of evolution over time based on the assumption that niches are saturated by accumulating species within a lineage, and therefore, describing scenarios where accumulated diversities play a role in the rates of lineage accumulations themselves. Finally, a delta model, which describes a time‐dependent model of trait evolution, where the effects that early versus late evolution in the tree have on the rates of trait diversification. This model returns a δ value which indicates whether recent evolution has been fast when δ > 1, or slow when δ < 1; Astudillo‐Clavijo, Arbour, & Lopez‐Fernandez, 2015; Hernández et al., 2013; Pincheira‐Donoso, Harvey, & Ruta, 2015). To compare the goodness of fit of these alternative models, we employed an Akaike information criterion (AIC) approach. We provide values reported as AICc (bias‐corrected version of AIC) and ΔAICc (the difference between each model and the best model). The best‐fitted model is determined by identifying the lowest AICc score, which equals 0 (Pincheira‐Donoso et al., 2015). All model analyses and fitting were performed with the R package “geiger” (Harmon, Weir, Brock, Glor, & Challenger, 2008).

We subsequently investigated whether the chemical compounds have evolved around an optimum value (i.e., whether their diversification has been influenced by stabilizing selection promoting convergences of the traits around one or more peaks on a “Simpsonian landscape”), by employing the R package “surface” (Ingram, Mahler, & Hansen, 2013; Mahler, Ingram, Revell, & Losos, 2013). The surface method fits an adaptive radiation model in which lineages on the studied phylogeny may experience convergent shifts toward adaptive optima on the above‐mentioned macro‐evolutionary Simpsonian landscape. Importantly, this model does not assume whether some lineages correspond to particular optima. Based on an OU model in which all species are pulled against a single adaptive optimum in morphospace, surface employs a stepwise model‐selection approach based on AICc, which allows for identification of the best model and the numbers and positions of adaptive peaks (i.e., trait “regimes”), and hence, for convergence toward these optima over evolutionary time (Ingram et al., 2013; Pincheira‐Donoso et al., 2015).

Finally, we used the amount of each compound to model their relative disparity across linages. We performed disparity‐through‐time (DTT) analyses. This analysis firstly calculates the average disparity for each trait over time (Hipsley, Miles, & Muller, 2014; Ingram, 2015; Jonsson, Lessard, & Ricklefs, 2015; Pincheira‐Donoso et al., 2015; Slater, Price, Santini, & Alfaro, 2010). DTT analyses compare the observed disparity values with those expected under a BM model after 10,000 simulations across phylogeny. Subsequently, the average body size disparity obtained from both the real and the simulated data is plotted against the age of the nodes to calculate the morphological disparity index (MDI). This index quantifies the overall difference in relative disparity for the studied trait among and within subclades (i.e., differences in the range of variation) compared with the expectation under the null BM model of evolution (Slater et al., 2010). More specifically, negative MDI scores indicate lower‐than‐expected trait relative disparity under BM (i.e., low average subclade relative disparity), which indicates that the majority of disparity occurs among subclades and thus that they occupy smaller and more isolated areas of the morphospace. Positive MDI values indicate that relative disparity among subclades shows a stronger overlap in morphospace(Pincheira‐Donoso et al., 2015). We conducted DTT analyses using the R package “geiger” (Harmon et al., 2008). In addition, we used the R package “phytools” (Revell, 2012) to project the phylogeny within morphospace defined by time on *x*‐axis (My since the root) and the relative abundance of each compound on *y*‐axis. Also, we reconstructed the relative abundance of each compound for ancestral species in the tree (Revell & Freckleton, 2013).

## 3 Results

### 3.1 Relative amount of species chemical compounds in the study

**Figure 1:**
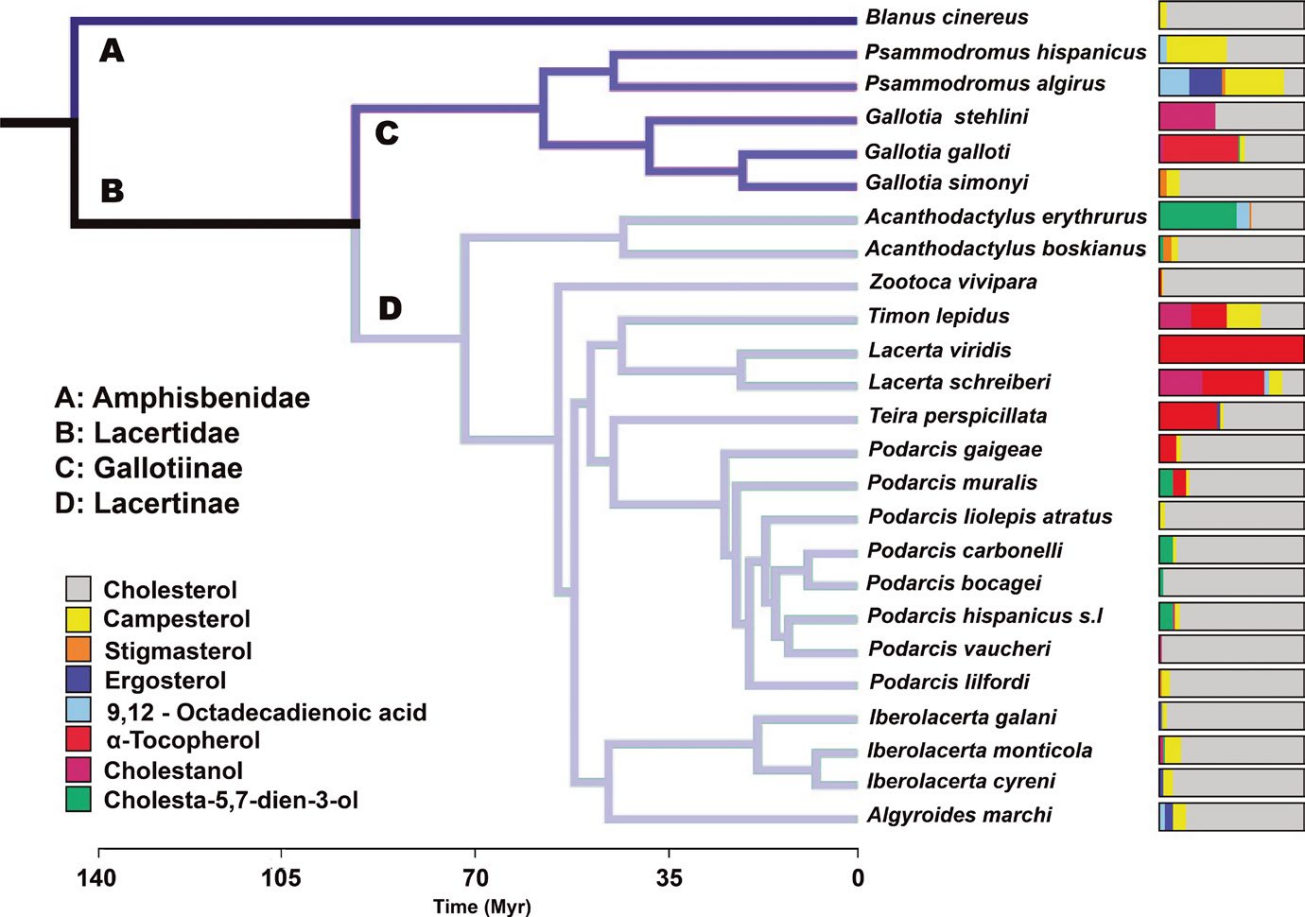
Phylogenetic relationship between analyzed species. Color bands show relative amounts of each compound with respect to the others for chemical secretions of the different analyzed species

Our analyses reveal that cholesterol is the predominant compound in our species (73.61%), followed by α‐tocopherol (9.96%), campesterol (7.61%), cholestanol (3.98%), cholesta‐5,7‐dien‐3‐ol (1.98%), 9,12‐octadecadienoic acid (1.28%), ergosterol (1.19%), and stigmasterol (0.39%; Figure 1). All these values, however, vary in the overall chemical profile description of each species (see Table S1 for details).

### 3.2 Tempo and mode of compound diversification

Our analyses comparing the four models of evolution performed among chemical compounds revealed substantial variation in the evolutionary trajectories followed by each of them during the clade's phylogenetic history (Table 1). While the analyses identified the stabilizing selection (OU model) as the best approximation to describe diversification for cholesterol, α‐tocopherol, and cholesta‐5,7‐dien‐3‐ol, the BM model best described the evolution of the remaining compounds. In addition, the three compounds for which the OU model was selected showed different numbers of local adaptive peaks on the Simpsonian landscape. More specifically, while we found a single optimum value for cholesterol (32.7%), six optimal values for α‐tocopherol (0.07%, 6.91%, 18.73%, 28.2%, 32.08%, and 37.2%) and cholesta‐5,7‐dien‐3‐ol (0.02%, 1.26%, 2.27%, 4.48%, 7.5%, and 8.5%) were identified by the surface analyses.

**Table 1:** Evolutionary diversification models of chemical compounds

Linage	Model	Model parameters	β	LogL	AICc	ΔAICc
Cholesterol	BM	–	2187.89	−117.59	239.72	2.52
	**OU**	**α = 2.72**	**4000.40**	**−115.02**	**237.20**	**0.00**
	EB	α = −0.00	2187.89	−117.58	242.32	5.12
	Delta	δ = 2.99	941.34	−115.63	238.40	1.20
Campesterol	**BM**	**–**	**67.53**	**−74.11**	**152.77**	**0.00**
	OU	α = 0.02	67.99	−74.11	155.37	2.60
	EB	α = −0.00	67.53	−74.11	155.37	2.60
	Delta	δ = 1.63	47.35	−73.97	155.10	2.33
Stigmasterol	**BM**	**–**	**0.57**	**−14.42**	**33.40**	**0.00**
	OU	α = 2.72	1.17	−13.30	33.75	0.34
	EB	α = −0.00	0.57	−14.42	36.00	2.60
	Delta	δ = 2.99	0.26	−13.25	33.65	0.24
Ergosterol	**BM**	**–**	**8.46**	**−48.14**	**100.84**	**0.00**
	OU	α = 0.00	8.46	−48.15	103.44	2.60
	EB	α = −0.21	10.12	−48.15	103.43	2.59
	Delta	δ = 2.05	5.12	−47.91	102.97	2.13
9,12‐Octadecanoic acid	**BM**	**–**	**5.40**	**−42.53**	**89.61**	**0.00**
	OU	α = 0.00	5.40	−42.53	92.21	2.60
	EB	α = −4.79	230.55	−41.57	90.30	0.68
	Delta	δ = 0.99	5.43	−42.53	92.21	2.60
Tocopherol	BM	–	417.73	−96.89	198.33	0.38
	**OU**	**α = 2.71**	**832.20**	**−95.40**	**197.95**	**0.00**
	EB	α = −0.00	417.74	−96.89	200.93	2.98
	Delta	δ = 2.99	187.65	−95.47	198.09	0.14
Cholestanol	**BM**	**–**	**88.21**	**−77.45**	**159.45**	**0.00**
	OU	α = 2.71	179.63	−76.23	159.62	0.17
	EB	α = −0.00	88.21	−77.45	162.05	2.60
	Delta	δ = 2.99	40.26	−76.23	159.61	0.16
Cholesta‐5,7‐dien‐3‐ol	BM	–	25.82	−62.09	128.74	5.37
	**OU**	**α = 2.71**	**42.13**	**−58.11**	**123.37**	**0.00**
	EB	α = −0.00	25.82	−62.09	131.33	7.97
	Delta	δ = 2.99	10.55	−59.49	126.13	2.76

Data values are based on comparing four evolutionary models. Fitted models are Brownian motion (BM), Ornstein–Uhlenbeck (OU), early‐burst (EB), and delta. Best fit of models based on (delta) bias**‐**corrected Akaike information criteria (AICc).

**Figure 2:**
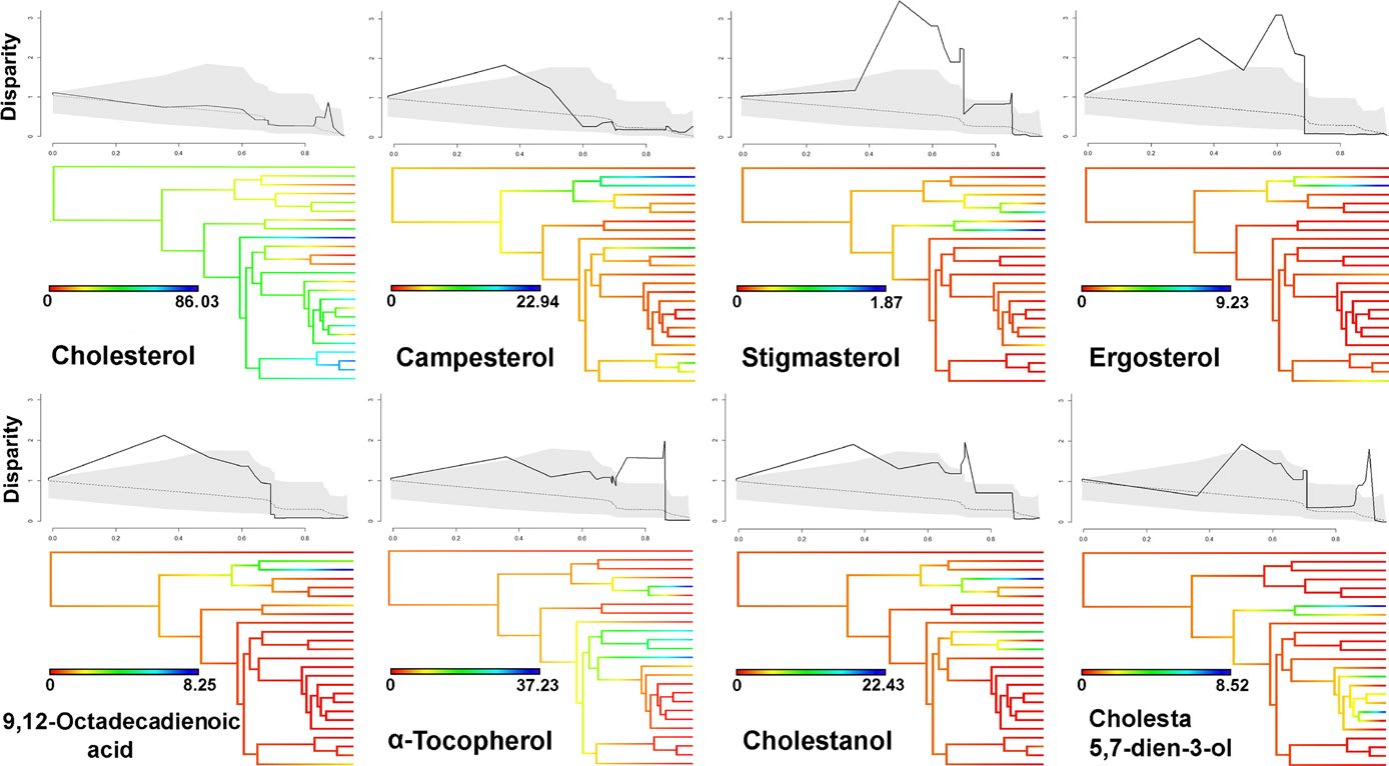
Tempo and mode of evolutionary diversification of proportions of chemical compounds in secretions of lizards. The top plot shows mean subclade disparity through time (DTT) showing proportion of time from taxon origin to present (*x*‐axis) for lizards chemical compounds (lower solid line) compared with the median subclade DTT of phenotypic evolution under a BM model (dashed line). The gray band shows the 95% DTT range for the simulated data. Model is based on 10,000 simulations. The phylogenetic tree shows a maximum‐likelihood ancestral trait reconstruction of each compound across phylogeny

**Figure 3:**
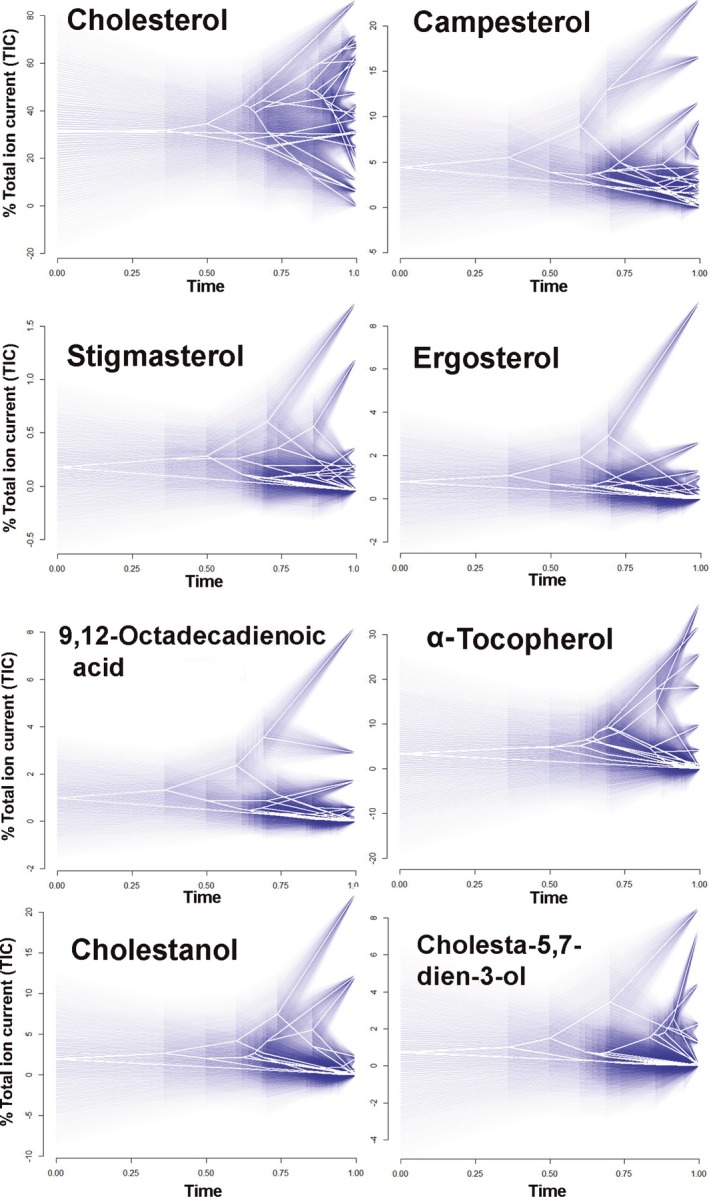
Chemical compounds evolution in lizards. The graph provides a morphospace projection of each chemical defined by relative time since the origin clade to present (*x*‐axis) and compound proportions (*y*‐axis), which state has been estimated using likelihood approach. The degree of uncertainty is indicated by increasing transparency of the plotted blue lines around the point estimates with the entire range showing the 95% confidence interval

The DTT analyses revealed positive MDI values in all compounds (i.e., higher values than expected under BM model). However, the evolutionary trajectories varied considerably among compounds. While campesterol (MDI = 0.31), stigmasterol (MDI = 0.84), ergosterol (MDI = 0.81), 9, 12‐octadecadienoic acid (MDI = 0.51), α‐tocopherol (MDI = 0.42), and cholestanol (MDI = 0.58) showed initial steep increases in relative disparity (in some cases slightly above the 95% CI), relative disparity of cholesterol (MDI = 0.04) and cholesta‐5,7‐dien‐3‐ol (MDI = 0.31) decreased early during the clade's history (Figure 2). In fact, the cholesterol DTT plot reflects an overall tendency to decrease over time. Only in the more recent segment of the clade's phylogenetic history (around. 6 Myr), relative disparity increases slightly above the upper limit of the 95% CI. Prominent increases and decreases are observed in the relative disparity of stigmasterol, ergosterol, α‐tocopherol, cholestanol, and cholesta‐5,7‐dien‐3‐ol plots, between 140 and 10 Mya, sometimes exceeding the 95% CI (Figure 2). Finally, diversification of each compound across the phylogeny shows strong morphospace overlapping in the ancestral trajectories of their evolution (Figure 3).

## 4 Discussion

Our study provides the first analysis investigating the phylogenetic macro‐evolutionary diversification dynamics of chemical signals employed by lizards during sexual communication and social communication. Our results reveal a clear pattern of heterogeneous tempo and mode of evolutionary diversification among different compounds within each species' chemosignals and across species. That is, we show that the chemical compounds might follow a “mosaic” (or “modular”) mode of evolutionary diversification where changes in some chemicals do not necessarily influence the others in coordination. Consequently, our findings have two major implications. Firstly, given that both the presence/absence, as well as the relative abundance, of some compounds might diversify independent from the other components of the scents, we suggest that chemical signals could embody a complex network of elements with potentially high and dynamic evolutionary lability given the weak degree of “chemical correlation” observed among them. And therefore, second, we suggest that selection is likely to have shaped the overall conformation of the chemical scents by exerting asymmetric effects on each chemical compound, thus promoting asymmetric rates of diversification that make this complex mosaic pattern emerge. The effect of selection on compounds is expected to be associated with the functional or structural role that each of them play in signal efficiency in different environments (e.g., social, ecological, or climatic; Baeckens, Huyghe, Palme, & Van Damme, 2016; Martín & López, 2015). Indeed, our ancestral reconstruction analyses reveal that multiple episodes of phenotypic shifts have occurred during different periods along the phylogeny (Figure 2). Interestingly, our analyses studying different models of evolution show that the two major compounds, cholesterol and α‐tocopherol, both of which have been assigned structural properties (Martín & López, 2014; Weldon et al., 2008), were found to have been shaped by stabilizing selection (OU model). The evolutionary pattern of cholesterol proportions revealed by ancestral reconstruction analyses shows episodes in which some species experienced changes toward reduced proportions or even total disappearance of the compound. Given its structural function, the diversification of the relative abundance of cholesterol in chemical secretions might be subject to selective pressures exerted by environment. Intriguingly, the evolution of cholesterol seems to follow an inverted pattern with respect to α‐tocopherol in some *Lacerta sensu lato* species (e.g., genus *Lacerta*,* Timon* and *Zootoca*; Figure 2). However, despite our results revealing heterogeneous trajectories of diversification across compounds and across species, we also observed that, as it would be expected, some of the compounds show a degree of coordinated evolution, revealing patterns of parallel evolution across lineages. This fact would be especially expected in components such as cholesterol, cholesta‐5,7‐dien‐3‐ol, ergosterol, and α‐tocopherol, given that their relative proportions in the scents are mediated by physiological trade‐offs arising from the high costs involved in their production (Kopena et al., 2011; Martín & López, 2006d, 2007, 2012, 2015). Therefore, physiological costs to allocate high abundances of some compounds to secretions could influence the allocation of high amounts of other chemicals, thus leading to the emergence of the above‐mentioned trade‐offs as the basis for some form of “chemical conflict” among compounds.

Likewise, our model‐selection analyses based on the DTT simulations reveal that the patterns and rates of evolutionary diversification among compounds differ substantially across species (Figures 2 and 3). These findings lead us again to reinforce the hypothesis that the chemical network which all compounds are part of is evolutionarily labile given that different factors (i.e., different selection pressures) can target different compounds rather independently to shape the optimal relative proportion of the chemical components needed to make the signal efficient and as cost‐effective as possible in each different environment. For example, multiples evidences have shown that chemical signal composition might vary according to different climatic conditions where lizards inhabit as an adaptive response to maximize the efficiency of chemical signals (Escobar et al., 2003; Martín, López, Garrido, Pérez‐Cembranos, & Pérez‐Mellado, 2013; Martín et al., 2015). Likewise, it has been shown that the relative abundance of some compounds, such as cholesterol and α‐tocopherol, can experience adaptive variations across species of lizards as a function of variation in the climatic conditions they are exposed to (Gabirot, Lopez, & Martín, 2012). However, not only structural compounds play key roles in the efficiency of signal production and delivery in lizard. Some steroids (e.g., cholesterol, campesterol, stigmasterol, and cholestanol), as well as α‐tocopherol and fatty acids (e.g., 9,12‐octadecadienoic acid), have been associated with lizard health conditions (Martín & López, 2014, 2015; Weldon et al., 2008). Additionally, steroids that act as vitamin precursors (e.g., ergosterol of vitamin D_2_ and cholesta‐5,7‐dien‐3‐ol of vitamin D_3_) are also believed to play important roles in signaling the health condition of the sender, mostly males (Martín & López, 2015). Thus, these compounds that provide information about “quality” of the signaler have increasingly been suggested to generate variance in the chances of getting access to sexual mates among males during both male–male interactions (Martín et al., 2007) and female mate choice (Martín & López, 2000, 2006d). Therefore, as suggested by previous studies (Symonds & Elgar, 2008), the combination between the facts that chemical compounds have a tendency to diversify independently from each other, that climatic factors can influence their adaptation, and the crucial roles that many of the components play in fitness‐linked activities, such as competition over mates, reinforces our view that chemical signals are potentially highly evolutionarily label. Collectively, the findings presented in this paper combined with previous research investigating the signaling roles of scents provide a series of lines of evidence highlighting the importance in increasing the impetus in investigating chemical signals not only in the traditional context of behavioral ecology, but also under a macro‐evolutionary perspective.

Previous studies have shown the key role of animal signals during species diversification, which can operate as drivers influencing diversification, thus playing roles during the causes and the consequences of their evolution (Maynard Smith & Harper, 2003). Our study shows different evolutionary patterns in relevant compounds found in sexual chemical signals. To date, the evolutionary trajectories of the presence and abundance of these compounds in chemical signals have remained fundamentally neglected, and thus, our study provides a starting baseline to highlight the need to continue with studies of a similar nature, but replicated across other organisms. Ecological pressures responsible for natural selection operating on signal efficiency are likely to influence the abundance of chemical components.

Our study is the first to investigate the macro‐evolutionary diversification of the chemical signals and their specific components in an explicit comparative context, and thus, we are aware that our results may suffer from limitations, especially given that we are making general inferences based on a limited number of species from the same clade and with a focus on some compounds chosen based on their known roles during signal production and delivery. However, until now, the numbers of species for which data on the chemical composition of their signals are available, as well as the compounds whose functionality has been studied, are highly limited and therefore a rather intrinsic limitation for this type of studies. Further research with larger numbers of species and compounds is therefore an important need to expand our understanding of the evolution of this dimension of animal communication, especially in lineages like lizards, in which chemical signals have been suggested to replace and eclipse the role of quantitative traits that operate as efficient signals in other lineages. Despite the limitations of this study, our findings provide a first and replicated evolutionary overview that should be considered in developing future evolutionary and ecological hypotheses centered around chemical communication.

## Conflict of Interest

None declared.
