# Supplementary material for: Heterogeneous tempo and mode of evolutionary diversification of compounds in lizard chemical signals
Source: Ecol Evol. 2017 Jan 29;7(4):1286–96. doi: 10.1002/ece3.2647 (PMC5306189; doi:10.1002/ece3.2647)
Supplement: Supplementary file 1 [file ECE3-7-1286-s001.doc]

**Table S1: Relative amount of eight lipophilic compounds found in male's femoral secretions of 25 lacertoid species.**

| Species | Cholesterol | Campesterol | Stigmasterol | Ergosterol | 9,12-Octadecadienoic acid | α-Tocopherol | Cholestanol | Cholesta-5,7-dien-3-ol | Reference |
| --- | --- | --- | --- | --- | --- | --- | --- | --- | --- |
| *Blanus cinereus* | 30.49 | 1.42 | 0.00 | 0.00 | 0.00 | 0.00 | 0.00 | 0.00 | López and Martín 2005c |
| *Psammodromus hispanicus* | 31.50 | 22.94 | 0.00 | 0.00 | 2.90 | 0.00 | 0.00 | 0.00 | López and Martín 2009 |
| *Psammodromus algirus* | 6.02 | 16.57 | 0.24 | 9.23 | 8.25 | 0.17 | 0.00 | 0.04 | Martín and López 2006a |
| *Gallotia stehlini* | 11.54 | 0.00 | 0.18 | 0.00 | 0.00 | 0.00 | 22.43 | 0.00 | * |
| *Gallotia galloti* | 31.00 | 2.91 | 0.20 | 0.05 | 0.50 | 37.20 | 2.21 | 0.00 | García-Roa et al. Submitted |
| *Gallotia simonyi* | 24.94 | 2.41 | 1.31 | 0.00 | 0.00 | 0.00 | 0.00 | 0.00 | Martín et al. 2015 |
| *Acanthodactylus erythrurus* | 6.00 | 0.06 | 0.00 | 0.00 | 1.73 | 0.04 | 0.00 | 8.52 | López and Martín 2005a |
| *Acanthodactylus boskianus* | 30.78 | 1.58 | 1.87 | 0.00 | 0.00 | 0.00 | 0.00 | 1.26 | Khannoon et al. 2011 |
| *Zootoca vivipara* | 86.03 | 0.78 | 0.00 | 0.00 | 0.00 | 1.16 | 0.00 | 0.00 | Gabirot et al. 2008 |
| *Timon lepidus* | 10.79 | 11.65 | 0.23 | 0.00 | 0.00 | 27.90 | 12.30 | 0.00 | Martín and López 2010 |
| *Lacerta viridis* | 0.00 | 0.00 | 0.00 | 0.00 | 0.00 | 25.63 | 0.00 | 0.00 | Kopena et al. 2009 |
| *Lacerta schreiberi* | 6.43 | 3.90 | 0.00 | 0.00 | 0.56 | 18.74 | 12.30 | 0.00 | López and Martín 2006 |
| *Scelarcis perspicillata* | 47.42 | 0.50 | 0.00 | 1.29 | 0.00 | 32.08 | 0.00 | 0.05 | * |
| *Podarcis gaigeae* | 14.50 | 2.39 | 0.13 | 0.49 | 0.07 | 7.44 | 0.00 | 0.17 | Runemark et al. 2011 |
| *Podarcis muralis* | 18.55 | 0.60 | 0.19 | 0.13 | 0.12 | 6.40 | 0.00 | 4.49 | Martín et al. 2008; Pellitteri-Rosa et al. 2014 |
| *Podarcis liolepis* | 63.38 | 2.66 | 0.00 | 0.09 | 0.18 | 0.00 | 0.00 | 0.00 | Gabirot et al. 2010 |
| *Podarcis carbonelli* | 20.20 | 0.38 | 0.00 | 0.00 | 0.01 | 0.00 | 0.00 | 2.27 | García-Roa et al. 2016 |
| *Podarcis bocagei* | 42.36 | 0.00 | 0.00 | 0.00 | 0.00 | 0.00 | 0.00 | 1.26 | García-Roa et al. 2016 |
| *Podarcis hispanicus* | 64.36 | 2.71 | 0.25 | 0.01 | 0.07 | 0.00 | 0.56 | 7.51 | Martín and López 2006b; Gabirot et al. 2010; Gabirot et al. 2012 |
| *Podarcis vaucheri* | 13.43 | 0.00 | 0.00 | 0.00 | 0.00 | 0.04 | 0.00 | 0.00 | * |
| *Podarcis lilfordi* | 64.01 | 4.24 | 0.42 | 0.00 | 0.01 | 0.00 | 0.00 | 0.00 | Martín et al. 2013 |
| *Iberolacerta galani* | 61.95 | 1.80 | 0.00 | 0.13 | 0.00 | 0.00 | 0.00 | 0.00 | * |
| *Iberolacerta monticola* | 73.64 | 9.68 | 0.01 | 0.43 | 0.00 | 0.00 | 2.02 | 0.00 | López et al. 2009 and unpublished data* |
| *Iberolacerta cyreni* | 67.01 | 5.33 | 0.00 | 1.02 | 0.58 | 0.00 | 0.00 | 0.14 | López and Martín 2005b |
| *Algyroides marchi* | 59.07 | 4.48 | 0.00 | 2.62 | 1.72 | 0.00 | 0.00 | 0.00 | * |

The relative amount of each component is determined as the percent of the total ion current (TIC) and reported as the average. (*) Species with no previous description of the eight chemical compounds. Samples are from adult males of the species *Gallotia stehlini* (27º 44' N, 15º 35' W; n=6), *Scelarcis perspicillata* (40º 00' N, 03º 52' E; n=5), *Podarcis vaucheri* (35º 10’ N, 2º 25’ W; n=8), *Iberolacerta galani* (42º 24’ N, 6º 24’ W; n=10), *I. monticola* (43º 19’ N, 8º 15 W; n=13) and *Algyroides marchi* (38º 20’N, 1º 38’ W; n=6).

**References**

Gabirot, M., A. M. Castilla, P. López, and J. Martín. 2010. Differences in chemical signals may explain species recognition between an island lizard, *Podarcis atrata*, and related mainland lizards, *P. hispanica*. ‎Biochem. Syst. Ecol. 38:521-528.

Gabirot, M., P. López, and J. Martín. 2012. Interpopulational variation in chemosensory responses to selected steroids from femoral secretions of male lizards, *Podarcis hispanica*, mirrors population differences in chemical signals. Chemoecology 22:65-73.

Gabirot, M., P. Lopez, J. Martín, M. De Fraipont, B. Heulin, B. Sinervo, and J. Clobert. 2008. Chemical composition of femoral secretions of oviparous and viviparous types of male common lizards *Lacerta vivipara*. Biochem. Syst. Ecol. 36:539-544.

García-Roa, R., C. Cabido, P. López, and J. Martín. 2016. Interspecific differences in chemical composition of femoral gland secretions between two closely related wall lizard species, *Podarcis bocagei* and *Podarcis carbonelli*. Biochem. Syst. Ecol. 64:105-110.

Khannoon, E. R., B. Flachsbarth, A. El-Gendy, K. Mazik, J. D. Hardege, and S. Schulz. 2011. New compounds, sexual differences, and age-related variations in the femoral gland secretions of the lacertid lizard *Acanthodactylus boskianus*. Biochem. Syst. Ecol. 39:95-101.

Kopena, R., P. López, and J. Martín. 2009. Lipophilic compounds from the femoral gland secretions of male Hungarian green lizards, *Lacerta viridis*. Z. Naturforsch. C Bio. Sci. 64:434-440.

López, P. and J. Martín. 2005a. Age related differences in lipophilic compounds found in femoral gland secretions of male spiny-footed lizards, *Acanthodactylus erythrurus*. Z. Naturforsch. C Bio. Sci. 60:915-920.

López, P. and J. Martín. 2005b. Chemical compounds from femoral gland secretions of male Iberian rock lizards, *Lacerta monticola cyreni*. Z. Naturforsch. C Bio. Sci. 60:632-636.

López, P. and J. Martín. 2005c. Intersexual differences in chemical composition of precloacal gland secretions of the amphisbaenian *Blanus cinereus*. J. Chem. Ecol. 31:2913-2921.

López, P. and J. Martín. 2006. Lipids in the femoral gland secretions of male Schreiber’s green lizards, *Lacerta schreiberi*. Z. Naturforsch. C Bio. Sci. 61:763-768.

López, P. and J. Martín. 2009. Lipids in femoral gland secretions of male lizards, *Psammodromus hispanicus*. Biochem. Syst. Ecol. 37:304-307.

López, P., P. L. Moreira, and J. Martín. 2009. Chemical polymorphism and chemosensory recognition between *Iberolacerta monticola* lizard color morphs. Chem. Senses 34:723-731.

Martín, J., L. Amo, and P. López. 2008. Parasites and health affect multiple sexual signals in male common wall lizards, *Podarcis muralis*. Naturwissenschaften 95:293-300.

Martín, J. and P. López. 2006a. Age-related variation in lipophilic chemical compounds from femoral gland secretions of male lizards *Psammodromus algirus*. Biochem. Syst. Ecol. 34:691-697.

Martín, J. and P. López. 2006b. Interpopulational differences in chemical composition and chemosensory recognition of femoral gland secretions of male lizards *Podarcis hispanica*: implications for sexual isolation in a species complex. Chemoecology 16:31-38.

Martín, J. and P. López. 2010. Multimodal sexual signals in male ocellated lizards *Lacerta lepida*: vitamin E in scent and green coloration may signal male quality in different sensory channels. Naturwissenschaften 97:545-553.

Martín, J., P. López, M. Garrido, A. Pérez-Cembranos, and V. Pérez-Mellado. 2013. Inter-island variation in femoral secretions of the Balearic lizard, *Podarcis lilfordi* (Lacertidae). Biochem. Syst. Ecol. 50:121-128.

Martín, J., A. Martínez-Silvestre, P. López, A. Ibáñez, M. Á. Rodríguez-Domínguez, and I. Verdaguer. 2015. Lipophilic compounds in femoral secretions of males and females of the El Hierro giant lizard *Gallotia simonyi* (Lacertidae). Biochem. Syst. Ecol. 61:286-292.

Pellitteri-Rosa, D., J. Martín, P. López, A. Bellati, R. Sacchi, M. Fasola, and P. Galeotti. 2014. Chemical polymorphism in male femoral gland secretions matches polymorphic coloration in common wall lizards (*Podarcis muralis*). Chemoecology 24:67-78.

Runemark, A., M. Gabirot, and E. Svensson. 2011. Population divergence in chemical signals and the potential for premating isolation between islet‐and mainland populations of the Skyros wall lizard (*Podarcis gaigeae*). J. Evol. Biol. 24:795-809.
